# COVID-19 vaccine literacy in patients with systemic autoimmune diseases

**DOI:** 10.1007/s12144-022-02713-y

**Published:** 2022-01-18

**Authors:** María Correa-Rodríguez, Blanca Rueda-Medina, José-Luis Callejas-Rubio, Raquel Ríos-Fernández, Javier de la Hera-Fernández, Norberto Ortego-Centeno

**Affiliations:** 1 Department of Nursing, Faculty of Health Sciences, University of Granada, Granada, Spain; 2 Systemic Autoimmune Diseases Unit, San Cecilio University Hospital, Granada, Spain; 3 Present Address: Instituto de Investigación Biosanitaria Ibs.GRANADA, Granada, Spain; 4 School of Medicine, University of Granada, Granada, Spain

**Keywords:** Covid-19, Vaccination, Vaccine literacy, Attitudes, Beliefs, Systemic autoimmune diseases

## Abstract

COVID-19 related infodemic is a threat to the successful COVID-19 vaccination campaigns. This might be especially apparent for patients with autoimmune diseases since there is no data available about the balance between benefits and risks of the newly developed COVID-19 vaccines in this population. We aim (i) to evaluate vaccine literacy skills in a population of patients with systemic autoimmune diseases, (ii) to examine the potential associations between vaccine literacy skills and sociodemographic characteristics and (iii) to analyze the relationships between attitudes, perceptions and beliefs about current vaccinations and vaccine literacy skills and sociodemographic characteristics. A cross-sectional study was conducted among 319 patients with systemic autoimmune diseases (92% females; 49.5% of patients in the 31–50 years age category). The vaccine literacy levels were determined using the Health Literacy about Vaccination in adulthood in Italian (HLVa-IT). Sociodemographic characteristics including gender, age, country and area of residence, civil status, socioeconomic status, educational attainment and occupational status were evaluated. The mean vaccine literacy functional and interactive-critical scores were 2.59 ± 0.74 and 3.07 ± 0.60, respectively. The vaccine literacy interactive-critical score was higher in females than in males (p = 0.048). Interactive-critical scores were associated with the area of residence, civil status and socioeconomic status, with the highest score in urban area of ≥ 100.000 inhabitants (p = 0.045), in widow patients (p = 0.023) and in patients with high socioeconomic status (p = 0.018). Significant differences were observed between the different education levels, for both the functional and the interactive-critical scores (p = 0.002 and p < 0.001, respectively), the highest score was observed in patients who completed a university degree. The level of vaccine literacy for functional and interactive-critical scales were medium. Area of residence, civil status and socioeconomic status represented determinants of vaccine literacy interactive-critical scale. Educational attainment also contributes to vaccine literacy functional scale. Insight into these factors is required to ensure an optimal vaccine literacy level in patients with autoimmune diseases.

**Supplementary Information:**

The online version contains supplementary material available at 10.1007/s12144-022-02713-y.

## Introduction

Coronavirus disease 2019 (COVID-19), a pandemic caused by the novel coronavirus, is accompanied by the generation of a lot of misinformation, rumours and half‐backed conspiracy theories from several sources (The Lancet Infectious Diseases, 2020). The relentless flood of COVID-19 information from unfiltered channels such as social media is often conflicting or false leading to confusion in the population (Islam et al., 2020; Pavela Banai et al., 2021). In addition to fast and diverse, the information is also continually changing (Rovetta & Bhagavathula, 2020).

### COVID-19-Related Infodemic

The incessant COVID-19 information may lead to health information overload since the level of information is higher than individuals’ information processing capacity (Rathore & Farooq, 2020). Thus, there is a massive infodemic with population receiving vast quantities of information, much of which is not scientifically correct (Brailovskaia, Miragall, Margraf, Herrero, & Baños, 2021; Naeem & Bhatti, 2020). The overwhelming information can have unfavourable effects on the management of the COVID-19 pandemic since the general population may find difficult to differentiate between what are facts, and what are opinions or biases (Mohammed et al., 2021). In fact, recent authors stated that fighting current infodemic is now the new front in the COVID-19 battle since it poses a major problem for public health (Naeem & Bhatti, 2020).

### COVID-19 Vaccination Campaign

Vaccination has been proposed as the most cost-effective way of avoiding the health challenge of COVID-19 pandemic (Jaspal & Breakwell, 2021). However, the global vaccination campaign is threatened by infodemic (Farooq & Rathore, 2021). Anti-vaccine communities are planning strategies against COVID-19 vaccination campaign through different sources to disseminate fictions and rumours. This unverified and unscientific information may lead to disastrous consequences such as vaccine hesitancy (WHO, 2019). In this context, vaccine literacy has been defined as “not simply knowledge about vaccines, but also developing a system with decreased complexity to communicate and offer vaccines as sine qua none of a functioning health system” (Ratzan, 2011).

Additionally, it is essential to determine levels of acceptance of the COVID-19 vaccine as well as perceptions, beliefs and attitudes towards COVID-19 vaccine to identify the strategies that will support the engagement. Previous studies have examined public perceptions, behaviors and beliefs towards COVID-19 vaccine in general populations of Australia (Seale et al., 2021), Greece (Zampetakis & Melas, 2021), India (Kalam et al., 2021) or China (Wong et al., 2021). Cognitive biases and irrational beliefs might be critical to vaccination behaviors (Tanhan et al., 2020). Azarpanah et al. recently identified potential cognitive biases that might affect the vaccination decision-making process and nudge people toward vaccine hesitancy (Azarpanah et al., 2021). These authors proposed that cognitive biases should be considered in any plans and interventions to increase vaccine trust and acceptability, particularly for COVID-19 vaccines.

### Systemic Autoimmune Diseases and COVID-19

Patients with systemic autoimmune diseases have an increasing vulnerability to the COVID-19 infection (Saad et al., 2021). The heterogeneous nature of the systemic autoimmune diseases and the immunosuppressive therapy might lead to concerns of severe outcomes in these patients. Thus, the perception towards the vaccination and immunological response might vary compared to the general population (Ali et al., 2021; Gaur et al., 2021). The reservations to get vaccinated against COVID-19 might be especially apparent for these patients since there is no data available about the balance between benefits and risks of the newly developed COVID-19 vaccines in this population (Boekel et al., 2021; Eftimov et al. 2021).

COVID-19 related infodemic is also a threat to the successful COVID-19 vaccination campaigns for systemic autoimmune diseases patients. Therefore, it is important to assess patients´s abilities to collect and understand information regarding vaccination and evaluate the association between vaccine literacy skills and sociodemographic characteristics among patients with autoimmune diseases. In this way, factors influencing patients ‘decision to get vaccinated would be identified allowing the promotion of effective strategies to ensure a mass vaccination campaigns in patients with autoimmune diseases.

### Purpose of the Research

In this context, we hypothesized that vaccine literacy skills are associated with sociodemographic characteristics of patients with systemic autoimmune diseases and that, attitudes, perceptions and beliefs about current Covid-19 vaccinations might be linked to vaccine literacy skills and sociodemographic characteristics. Thus, the aims of this study were (i) to evaluate for the first time vaccine literacy skills in a population of patients with systemic autoimmune diseases, (ii) to examine the potential associations between vaccine literacy skills and sociodemographic characteristics and (iii) to analyze the relationships between attitudes, perceptions and beliefs about current vaccinations and vaccine literacy skills and sociodemographic characteristics.

## Methods

### Study Design

An anonymous online survey, which respondents could choose to complete or not, was conducted. The questionnaire was prepared, distributed, and collected by ‘LimeSurvey,’ an online service that creates web-based surveys. A web link collector generated the URL for the survey. Through this link, patients were able to access the survey and send their responses. Patients were recruited from an online systemic autoimmune disease association. The URL was posted to the public on the Associations’ Facebook page on May 8, 2021 until June 8, 2021. The study protocol was approved by Local Ethics Committee of University of Granada (2130/CEIH/2021).

### Participants

A total of 3369 patients ≥ 18 years of age who had been previously diagnosed with a systemic autoimmune disease by a professional and that were registered in this group were invited to answer the online questionnaire. Finally, a total of 319 patients with systemic autoimmune diseases were included in the study after giving written informed consent (92% females; 49.5% of patients in the 31–50 years age category). Respondents were required to provide honest answers, were not given any incentives for participation and could reply only once to the survey. They were informed that proceeding to the second page of the survey and completing the questionnaire constituted consent. ﻿

### Sociodemographic Characteristics

Firstly, each participant completed a structured questionnaire regarding sociodemographic characteristics including gender, age, country and area of residence, civil status, socioeconomic status, educational attainment and occupational status.

### Vaccine Literacy

The vaccine literacy levels were determined using the Health Literacy about Vaccination in adulthood in Italian (HLVa-IT) (Biasio et al., 2020). Five items of the questionnaire were aimed at assessing functional vaccine literacy and nine items evaluated interactive-critical vaccine literacy, according to Nutbeam’s definition (Nutbeam, 2000). From the psychometric point of view, functional VL questions were mainly about language, involving the semantic system, while the interactive-critical questions focused more on cognitive efforts, such as problem-solving and decision-making (Biasio et al., 2020). This questionnaire has already been validated for content and construct (Biasio et al., 2020). Each response was rated with a 4-point Likert scale (4 – never, 3 – rarely, 2 – sometimes, 1 – often, for the functional questions; 1 – never, 2 – rarely, 3 – sometimes, 4 – often, for the interactive- critical questions). The score was obtained from the mean value of the answers to each scale (range 1 to 4), a higher value corresponding to a higher vaccine literacy level.

### Attitudes, Perceptions and Beliefs About COVID-19 Vaccines

Attitudes and perceptions about COVID-19 vaccines and current vaccinations were assessed by questions measured by a nominal scale (Biasio et al., 2021a, 2021b). Moreover, eight statements used a 4-point Likert scale to evaluate participants’ beliefs about COVID-19 vaccines (Biasio, et al., 2021a, 2021b; Seale et al., 2021).

### Statistical Analysis

SPSS® Statistics version 21.0 (SPSS, Chicago, IL, USA) was used for all analyses. Continuous variables were presented as mean ± standard deviation and categorical variables as frequencies and percentages. ﻿To analyze the normality of the distribution of the variables (p > 0.05), we used the Kolmogorov–Smirnov test. ﻿All variables had non-normal distributions and therefore, the Mann–Whitney U test for continuous data and ﻿Fisher’s exact tests were used for data analysis. ﻿Also, Kruskal–Wallis test were used. P values of < 0.05 were considered statistically significant.

## Results

The sociodemographic characteristics of the study population are shown in Table 1. Most patients were females (92%), and about 50% of patients were in the 31–50 years age category. Most patients were living in Spain (81.1%) and in an urban area (80.4%). Almost half of the patients were married (48.3%) and had a middle (47.8%) or high socioeconomic status (45.8%). Moreover, the 45.5% of patients had completed a university degree and the 47.1% were currently working in private or public sectors. In the study cohort, the prevalence of systemic lupus erythematosus (SLE) was the highest (41.6%), subsequently followed by vasculitis (23.3%), antiphospholipid syndrome (7.9%), scleroderma (4.7%), Sjögren syndrome (3.9%), sarcoidosis (3.6%), rheumatoid arthritis (2.3%) and espondyloarthritis (0.8%).

**Table 1:** Sociodemographic characteristics of the overall study population and according to the vaccine literacy (VL) functional and interactive-critical skills

	Overall	VL functional score		VL interactive-critical score	
	N (%)	Mean (SD)	P value	Mean (SD)	P value
Gender					
Male	31 (8.0)	2.52 (0.91)	0.690	2.80 (0.69)	**0.048**
Female	356 (92.0)	2.59 (0.72)		3.09 (0.58)	
Age groups					
18–30	46 (11.9)	2.61 (0.75)	0.119	2.96 (0.66)	0.328
31–50	192 (49.5)	2.62 (0.71)		3.04 (0.63)	
51–65	129 (33.2)	2.47 (0.76)		3.14 (0.52)	
> 65	21 (5.4)	2.85 (0.75)		3.13 (0.55)	
Country of residence					
Spain	313 (81.1)	2.59 (0.75)	0.698	3.04 (0.58)	0.080
Other	73 (18.9)	2.55 (0.68)		3.19 (0.64)	
Area of residence					
Rural area	74 (19.6)	2.61 (0.67)	0.301	2.90 (0.57)	**0.045**
Urban area of < 100.000 inhabitants	133 (35.3)	2.67 (0.82)		3.08 (0.66)	
Urban area of ≥ 100.000 inhabitants	170 (45.1)	2.53 (0.69)		3.12 (0.55)	
Civil Status					
Single	88 (22.6)	2.55 (0.74)	0.472	3.03 (0.54)	**0.023**
Living-in unit	62 (15.9)	2.58 (0.72)		2.86 (0.67)	
Married	188 (48.3)	2.64 (0.75)		3.12 (0.59)	
Separated or divorced	44 (11.3)	2.45 (0.69)		3.13 (0.55)	
Widow	7 (1.8)	2.26 (0.96)		3.48 (0.33)	
Socioeconomic status					
Low	25 (6.4)	2.46 (0.86)	0.155	3.11 (0.55)	**0.018**
Middle	186 (47.8)	2.52 (0.70)		2.97 (0.63)	
High	178 (45.8)	2.67 (0.75)		3.16 (0.55)	
Educational attainment					
Elementary	31 (8.1)	2.30 (0.77)	**0.002**	2.66 (0.74)	** < 0.001**
High school	58 (15.1)	2.35 (0.75)		2.92 (0.61)	
Post-secondary school	118 (30.6)	2.53 (0.70)		2.95 (0.64)	
College	175 (45.5)	2.74 (0.72)		3.25 (0.46)	
Occupational status					
Worker in the private sector	109 (28.2)	2.66 (0.76)	0.527	3.00 (0.65)	0.204
Worker in the public sector	73 (18.9)	2.50 (0.74)		3.20 (0.40)	
Not working	84 (21.7)	2.50 (0.72)		2.99 (0.66)	
Retired	74 (19.1)	2.62 (0.76)		3.12 (0.60)	
Others	47 (12.1)	2.59 (0.74)		3.06 (0.57)	

VL: vaccine literacy

### Vaccine Literacy Score

In the overall study population, the mean vaccine literacy functional and interactive-critical scores were 2.59 ± 0.74 and 3.07 ± 0.60, respectively, out of a maximum of 4. The vaccine literacy interactive-critical score was higher in females than in males while the functional scores were 2.52 ± 0.91 and 2.59 ± 0.72, respectively (non-significant difference) (Table 1). Interactive-critical scores were associated with the area of residence, civil status and socioeconomic status, with the highest score in urban area of ≥ 100.000 inhabitants, in widow patients and in patients with high socioeconomic status. Regarding the relationship between vaccine literacy and educational attainment, significant differences were observed between the different education levels, for both the functional and the interactive-critical scores, the highest score was observed un patients who completed a university degree. The answers to each question on the health literacy skills about vaccination (HLVa) scale including vaccine literacy functional and interactive-critical skills are showed in Table 2.

**Table 2:** Levels of health literacy skills about vaccination (HLVa) including vaccine literacy (VL) functional and interactive-critical skills

l		N (%)				Mean (SD)
		Never	Rarely	Sometimes	Often	
VL functional skills	When reading or listening to information about future COVID-19 vaccines or current vaccines:					
	1. Did you find that the material as a whole (texts and/or images) was difficult to read?	66 (18.4)	124 (34.6)	143 (39.9)	25 (7.0)	2.65 (0.86)
	2.Did you find words you didn’t know?	31 (9.1)	94 (27.6)	174 (44.1)	42 (12.3)	2.33 (0.80)
	3. Did you find that the texts were difficult to understand?	68 (19.9)	109 (32.0)	141 (41.3)	23 (6.7)	2.65 (0.87)
	4. Did you need much time to understand them?	55 (16.1)	99 (29.0)	142 (41.6)	45 (13.2)	2.48 (0.91)
	5. Did you or would you need someone to help you understand them?	98 (28.7)	119 (34.9)	96 (28.2)	28 (8.2)	2.84 (0.93)
VL interactive/critical skills	When looking for information about future COVID-19 vaccines or current vaccines:					
	6. Have you consulted more than one source of information?	22 (6.5)	30 (8.8)	106 (31.1)	183 (53.7)	3.32 (0.88)
	7. Did you find the information you were looking for?	11 (3.2)	18 (5.3)	177 (52.1)	134 (39.4)	3.28 (0.70)
	8. Did you understand the information found?	11 (3.2)	9 (2.6)	162 (47.6)	158 (46.5)	3.37 (0.69)
	9. Have you had the opportunity to use the information?	53 (15.6)	60 (17.6)	149 (43.8)	78 (22.9)	2.74 (0.98)
	10. Did you discuss what you understood about vaccinations with your doctor or other people?	79 (23.2)	64 (18.8)	118 (34.6)	80 (23.5)	2.58 (1.08)
	11. Did you consider whether the information collected was about your condition?	41 (12.1)	75 (22.1)	130 (38.2)	94 (27.6)	2.81 (0.97)
	12. Have you considered the credibility of the sources?	19 (5.6)	35 (10.3)	151 (44.3)	136 (39.9)	3.18 (0.83)
	13. Did you check whether the information was correct?	31 (9.1)	38 (11.1)	103 (30.2)	169 (49.6)	3.20 (0.96)
	14. Did you find any useful information to make a decision on whether or not to get vaccinated?	38 (9.6)	41 (12.1)	101 (29.7)	160 (47.1)	3.13 (1.01)

VL: vaccine literacy

### Attitudes and Perceptions About COVID-19 Vaccines and Behavior Toward Current Vaccines

Table 3 presented attitudes and perceptions about COVID-19 vaccines and behavior toward current vaccines, and their association with vaccine literacy scores and sociodemographic characteristics. Observed attitudes and perceptions on COVID- 19 vaccines were mostly positive, with affirmative responses between about 80% and 90% for all questions, except for questions n.3 *(‘Do you think they overlap, regardless of the production technique used?’*) and n.7 (*‘Would you pay a fee to be vaccinated? ‘*). Note that the 96.7% of patients had the intention to get vaccinated against COVID-19. Regarding behaviour toward current vaccines, the 62.2% of patients had been vaccinated against flu last season and almost half of the patients (48.2%) had been recently vaccinated and/or intend to be vaccinated soon against other infectious diseases.

**Table 3:** Attitudes and perceptions about COVID-19 vaccines and behavior toward current vaccines, and their association with vaccine literacy (VL) scores and sociodemographic characteristics

			VL functional score		VL interactive-critical score		Gender	Age groups	Country of residence	Area of residence	Civil status	Socioeconomic status	Educational attainment	Occupational status
About COVID-19 vaccines:	Answer	N (%)	Mean (SD)	P value	Mean (SD)	P value	P value	P value	P value	P value	P value	P value	P value	P value
1. Do you think the vaccines developed so far are safe?	Yes	275 (82.6)	2.66 (0.72)	**0.001**	3.12 (0.56)	** < 0.001**	0.458	0.087	0.470	0.134	0.513	**0.002 (7)**	0.263	**0.004 (8)**
	No	58 (17.4)	2.28(0.76)		2.76 (0.67)									
2. Do you think they are efficacious?	Yes	291 (87.7)	2.64 (0.72)	**0.013**	3.11 (0.55)	**0.004**	0.492	0.143	**0.010 (4)**	0.766	0.557	** < 0.001 (7)**	0.135	** < 0.001 (8)**
	No	41 (12.3)	2.31 (0.77)		2.71 (0.80)									
3. Do you think they overlap, regardless of the production technique used?	Yes	226 (68.1)	2.64 (0.71)	0.128	3.13 (0.56)	**0.003**	0.073	0.269	0.881	0.340	0.447	**0.003 (7)**	0.759	0.310
	No	106 (31.9)	2.50 (0.78)		2.91 (0.64)									
4.Do you intend to get vaccinated against COVID-19?	Yes	322 (96.7)	2.60 (0.74)	0.542	3.07 (0.58)	0.183	0.337	0.174	0.381	0.240	0.443	**0.019 (7)**	0.092	**0.042 (8)**
	No	11 (3.3)	2.47 (0.65)		2.68 (0.89)									
5. If you could, would you choose which vaccine to take?	Yes	270 (81.3)	2.60 (0.73)	0.901	3.05 (0.59)	0.741	0.721	**0.049 (1)**	**0.031 (5)**	0.860	0.332	0.975	0.960	**0.031 (9)**
	No	62 (18.7)	2.59 (0.76)		3.08 (0.63)									
6. Will the Government be able to offer the vaccine against COVID-19 for everyone for free?	Yes	330 (99.1)	2.59 (0.74)	0.362	3.05 (0.60)	**0.034**	0.620	0.390	0.465	0.164	0.872	0.073	0.497	0.344
	No	3 (0.9)	2.93 (0.50)		3.66 (0.22)									
7. Would you pay a fee to be vaccinated?	Yes	218 (65.7)	2.61 (0.73)	0.718	3.12 (0.56)	**0.016**	0.135	0.258	0.214	0.446	0.574	** < 0.001 (7)**	0.053	**0.002 (8)**
	No	114 (34.3)	2.58 (0.75)		2.94 (0.65)									
8. Should vaccination against COVID-19 be made mandatory for everyone?	Yes	269 (81.0)	2.61 (0.74)	0.550	3.04 (0.61)	0.139	0.146	0.176	**0.027 (5)**	0.211	0.781	0.521	0.135	0.374
	No	63 (19.0)	2.55 (0.71)		3.15 (0.53)									
9. Should vaccination against COVID-19 be made compulsory for the most at-risk groups?	Yes	290 (87.1)	2.59 (0.75)	0.936	3.05 (0.60)	0.748	0.446	0.722	0.133	0.721	0.819	0.304	0.195	0.562
	No	43 (12.9)	2.60 (0.71)		3.08 (0.55)									
10. Do you think children should be vaccinated too?	Yes	282 (84.9)	2.60 (0.73)	0.947	3.06 (0.60)	0.901	0.656	0.365	0.269	0.165	0.194	0.804	0.361	0.157
	No	50 (15.1)	2.60 (0.78)		3.07 (0.57)									
About current vaccines:			()		()									
11.Have you been vaccinated against flu last season?	Yes	207 (62.2)	2.57 (0.76)	0.416	3.06 (0.58)	0.770	0.844	**0.008 (2)**	** < 0.001 (4)**	0.787	**0.012 (6)**	**0.021 (7)**	0.306	**0.018 (10)**
	No	126 (37.8)	2.63 (0.70)		3.04 (0.63)									
12. Did you want to be vaccinated against the flu, but you couldn’t?	Yes	58 (17.5)	2.53 (0.68)	0.403	3.11 (0.67)	0.552	0.065	**0.009 (3)**	** < 0.001 (5)**	0.720	0.068	0.054	0.112	**0.006 (14)**
	No	274 (82.5)	2.61 (0.75)		3.05 (0.58)									
13. Have you been recently vaccinated and/or do you intend to be vaccinated soon against other infectious diseases, in addition to seasonal influenza and COVID-19?	Yes	160 (48.2)	2.65 (0.76)	0.214	3.14 (0.54)	**0.022**	0.984	0.552	**0.041 (5)**	0.569	0.680	0.327	0.250	0.332
	No	172 (51.8)	2.55 (0.71)		2.99 (0.63)									

(1) = More positive answers among patients aged 51–65 years

(2) = More positive answers among patients aged > 65 years

(3) = More negative answers among patients aged > 65 years

(4) = More positive answers among patients living in Spain

(5) = More positive answers among patients living in other countries

(6) = More positive answers among widow patients

(7) = More positive answers among patients with high socioeconomic status

(8) = More negative answers among patients not working

(9) = More negative answers among retired patients

(10) = More positive answers among patients not working

There were no associations between attitudes and perceptions about vaccines and vaccine literacy functional scores except for questions n.1 *(‘Do you think the vaccines developed so far are safe?’)* and n.2 *(‘Do you think they are efficacious?’)* where there were more positive answers in patients with higher vaccine literacy functions scores (Table 3). For vaccine literacy interactive-critical, there were significant associations for questions n.1 *(‘Do you think the vaccines developed so far are safe?’)*, n.2 *(‘Do you think they are efficacious?’)*, n.3 (*‘Do you think they overlap, regardless of the production technique used?’*), n.6 (*‘Will the Government be able to offer the vaccine against COVID-19 for everyone for free?’*), n.7 (*‘Would you pay a fee to be vaccinated?’*) and n.13 (*‘Have you been recently vaccinated and/or do you intend to be vaccinated soon against other infectious diseases, in addition to seasonal influenza and COVID-19?’*). In all these questions except for questions n.6, there were more positive answers in patients with higher vaccine literacy interactive critical scores.

### Beliefs About COVID-19 Vaccines

Beliefs about COVID-19 vaccines and their association with VL scores and sociodemographic characteristics are showed in Table 4. About 70% and 83% of patients disagreed completely (Likert score 4) with statements n.1 and n.2 1 *(‘I am not favorable to vaccines because they are unsafe’ and ‘There is no need to vaccinate because natural immunity exists’*). In contrast, the majority of respondents agreed completely (Likert score 1) for the statements n.3 *‘Vaccines are effective at preventing diseases’* (88.9%), n.4 *‘I generally do what my health care professional recommends’* (92.2%), n.5 *‘Getting myself vaccinated for COVID-19 would be a good way to protect myself against infection’* (87.7%), n.6 *‘My family and friends would probably think that getting a COVID-19 vaccine is a good idea’* (85.8%), n.7 *‘To protect public health, we should follow government guidelines about vaccines’* (81.0%), and n.8 *‘Patients with risk factors should be the first ones to get the COVID-19 vaccine when available’* (78.5%).

**Table 4:** Beliefs about COVID-19 vaccines and their association with vaccine literacy (VL) scores and sociodemographic characteristics

**How much do you agree with the following statements:**			**VL functional score**		**VL interactive-critical score**		**Gender**	**Age groups**	**Country of residence**	**Area of residence**	**Civil status**	**Socioeconomic status**	**Educational attainment**	**Occupational status**
	Answer	N (%)	Mean (SD)	P value	Mean (SD)	P value	P value	P value	P value	P value	P value	P value	P value	P value
**1. I am not favorable to vaccines because they are unsafe**	Totally agree	33 (9.9)	2.50 (0.71)	0.117	2.98 (0.67)	**0.003**	0.243	0.147	0.730	0.335	0.150	0.210	**0.044 (6)**	0.195
	Partially agree	36 (10.8)	2.36 (0.65)		2.90 (0.53)									
	Partially disagree	32 (9.6)	2.51 (0.77)		2.78 (0.62)									
	Totally disagree	232 (69.7)	2.65 (0.75)		3.13 (0.58)									
**2. There is no need to vaccinate because natural immunity exists**	Totally agree	12 (3.6)	2.58 (0.70)	0.802	3.02 (0.72)	0.477	0.611	0.429	0.849	**0.049 (3)**	0.695	0.861	0.851	0.533
	Partially agree	26 (7.8)	2.73 (0.74)		3.13 (0.57)									
	Partially disagree	19 (5.7)	2.57 (0.65)		2.86 (0.55)									
	Totally disagree	275 (82.8)	2.58 (0.75)		3.06 (0.60)									
**3. Vaccines are effective at preventing diseases**	Totally agree	295 (88.9)	2.57 (0.74)	0.235	3.07 (0.60)	0.054	0.664	0.956	0.543	0.157	0.671	0.783	0.889	0.814
	Partially agree	7 (2.1)	2.82 (0.46)		2.58 (0.53)									
	Partially disagree	5 (1.6)	2.28 (0.62)		2.62 (0.76)									
	Totally disagree	25 (7.5)	2.83 (0.75)		3.14 (0.54)									
**4. I generally do what my health care professional recommends**	Totally agree	306 (92.2)	2.59 (0.74)	0.613	3.07 (0.60)	0.419	0.412	0.880	0.762	0.213	0.445	0.182	0.698	0.987
	Partially agree	11 (3.3)	2.52 (0.73)		2.87 (0.46)									
	Partially disagree	5 (1.5)	2.52 (0.86)		2.71 (0.61)									
	Totally disagree	10 (3.0)	2.90 (0.79)		3.08 (0.48)									
**5. Getting myself vaccinated for COVID-19 would be a good way to protect myself against infection**	Totally agree	291 (87.7)	2.60 (0.72)	0.094	3.16 (0.49)	**0.002**	0.542	0.420	0.548	0.112	0.622	**0.040 (5)**	0.610	0.272
	Partially agree	21 (6.3)	2.35 (0.91)		2.64 (0.84)									
	Partially disagree	6 (1.8)	2.20 (0.61)		2.64 (0.78)									
	Totally disagree	14 (4.2)	2.90 (0.75)		3.09 (0.57)									
**6. My family and friends would probably think that getting a COVID-19 vaccine is a good idea**	Totally agree	285 (85.8)	2.59 (0.73)	0.309	3.08 (0.57)	0.055	0.223	0.717	0.622	**0.033 (4)**	0.182	0.301	0.765	0.490
	Partially agree	25 (7.5)	2.43 (0.82)		2.75 (0.85)									
	Partially disagree	11 (3.3)	2.74 (0.76)		3.00 (0.61)									
	Totally disagree	11 (3.3)	2.90 (0.86)		3.00 (0.61)									
**7. To protect public health, we should follow government guidelines about vaccines**	Totally agree	268 (81.0)	2.58 (0.72)	0.091	3.11 (0.49)	**0.019**	**0.002 (1)**	0.496	0.515	0.905	0.972	0.155	0.997	0.516
	Partially agree	33 (10.0)	2.40 (0.80)		2.74 (0.76)									
	Partially disagree	16 (4.8)	2.78 (0.72)		3.09 (0.46)									
	Totally disagree	14 (4.2)	2.94 (0.85)		3.09 (0.58)									
**8. Patients with risk factors should be the first ones to get the COVID-19 vaccine when available**	Totally agree	260 (78.5)	2.57 (0.74)	0.687	3.06 (0.60)	0.121	0.281	0.430	**0.027 (2)**	0.338	0.561	0.602	0.487	0.433
	Partially agree	48 (14.5)	2.66 (0.71)		3.06 (0.54)									
	Partially disagree	10 (3.0)	2.66 (0.92)		2.62 (0.85)									
	Totally disagree	13 (3.9)	2.76 (0.74)		3.17 (0.51)									

(1) = Greater agreement in females compared to males

(2) = Greater agreement in patients living in Spain compared to patients living in others countries

(3) = Greater agreement in patients living in rural areas compared to patients living in urban areas

(4) = Greater agreement in patients living in urban areas compared to patients living in rural areas

(5) = Greater agreement in patients with higher socioeconomic status compared to patients with lower socioeconomic status

(6) = Greater agreement in patients with elementary compared to patients with college educational attainment

## Discussion

Limited vaccine literacy is considered a component of vaccination convenience and a cause a low uptake of vaccines (Biasio, 2019). Currently, there is a growing volume of information regarding COVID-19 vaccines, especially contradictory information, that can have untoward effects on the general population and, especially, in autoimmune disease patients. In this study we identified that the vaccine literacy scores for functional and interactive-critical scales were medium (2.59 and 3.07, respectively) in patients with autoimmune diseases. Interactive-critical scores were associated with the gender, area of residence, civil status and socioeconomic status, and both functional and interactive-critical scores were related to educational attainment. By highlighting the factors associated with vaccine literacy in autoimmune diseases patients, the present study provides the ground for educational programs aimed at improving vaccine literacy.

The average vaccine literacy scores observed in the present study were lower than those observed from a recent study conducted in the general population (Biasio et al., 2021a, 2021b). Thus, Biasio et al. identified that the average for functional and interactive-critical scales were relatively high (2.92 and 3.27, respectively) in a Italian cohort (Biasio et al., 2021a, 2021b). This is unexpectedly since it has been reported that individuals with a history of a disease should be more interested in gaining information about diseases and improving their health literacy. However, similar to our findings, Biasio et al. observed that the average vaccine literacy functional score was lower than the interactive-critical one.

In this study, we identified the highest interactive-critical scores in females, in patients living in urban area of ≥ 100.000 inhabitants, in widow patients and in patients with high socioeconomic status. Previous studies assessing the general level of health literacy have also indicated that the level is significantly lower in men than in women (Clouston et al., 2017; Joveini et al., 2019; Oliffe et al., 2020). Also, noticeable differences were reported in health literacy between urban and rural populations. Recently, Wang et al. found that in rural areas they had higher odds to exhibit basic health literacy (Wang et al., 2020), supporting that the differences may be attributed to socioeconomic inequalities between these areas (Chen et al., 2019; Golboni et al., 2018). In contrast to our findings, Joveini et al. reported that widows/widowers and divorced/separated individuals had the lowest health literacy level in a population of Iranian Adults (Joveini et al., 2019). The contradictory findings might be attributed to differences in the target population and tools since all mentioned-studies assessed health literacy whereas in our study we specifically evaluated vaccine literacy. Also, the discrepancies should be explained by personal resources, as widows with higher income and good health have been shown to report higher levels of perceived competence for self-care (Utz et al., 2011). Additionally, widowed subjects who expected their spouse’s death may report higher levels of perceived competency after widowhood.

The relationship between socioeconomic status and vaccine literacy is not surprising and may be linked to education level, since subjects with university studies are more likely to have a higher socioeconomic status. Among the same line, recent research also identified economic hardship and education level as determinants of vaccine hesitancy (Bertoncello et al., 2020; Van Der Heide et al., 2013). In our study the highest scores for both functional and interactive-critical scales were found in patients who completed a university degree, supporting that a higher level of education is positively associated with a higher level of vaccine literacy. Reasonably, similar conclusions were reported from other studies (Joveini et al., 2019). It is logical to presume that improved education results in improved access to knowledge, health information-seeking behaviour and, in general, the opportunity to make sense of the information received (Mohammed et al., 2021). Moreover, education is recognized as the most critical determinant of health literacy (Joveini et al., 2019). Overall, these findings may serve as a warning light, since specific sociodemographic differences may be determinant to vaccine literacy and, consequently may condition the attitude and behaviour of autoimmune patients towards COVID-19 vaccination.

Observed attitudes and perceptions on COVID- 19 vaccines among autoimmune disease patients were mostly positive, with affirmative responses between about 80% and 90% for all questions, except for two questions. It is also especially relevant the high percentage (96.7%) of patients that have the intention to get vaccinated against COVID-19. Compared to our data, Boekel et al. reported remarkably lower proportions of vaccinations willingness in patients with autoimmune diseases (61%) (Boekel et al., 2021). Noteworthy, this difference might be explained by the recently published research reinforcing the safety and efficacy of the current vaccines that has been announce largely by the media. Furthermore, a relatively high percentage of patients (62.2%) stated that they been vaccinated against flu last season, and almost half of the patients (48.2%) had been recently vaccinated and/or intend to be vaccinated soon against other infectious diseases, in addition to seasonal influenza and COVID-19. These findings are expected since autoimmune disease patients are included as target group of flu vaccine recommendations (Urbinztondo Perdices & Borràs López, 2018). With regards to beliefs about vaccination, most patients disagreed completely with the negative statements about the relevance of vaccination whereas agreed completely with the positive statements.

This study has some limitations that must be acknowledged. Firstly, although the sample was large in size and provided a good cross-section of the population, selection bias cannot be excluded since patients with no Internet access or mobile phone literacy could not take part in this online survey. Nevertheless, in this study most patients were living in Spain (81.1%) and, according to the last annual market reported, 90% of Spaniards possess a cell phone, and for 91.5% of Spaniards, the cell phone is the device most frequently used to access the internet (Informe Descubriendo al Nuevo Consumidor, 2021). The questionnaire regarding attitudes, perceptions and beliefs about COVID-19 vaccines used in this study has not yet been validated. However, previous studies has used it previously (Biasio, et al., 2021a, 2021b; Seale et al., 2021). Despite its limitations, this preliminary study provides a first glance on the vaccine literacy level, attitude, perceptions, behavior in patients with autoimmune diseases. Also, this is the first study which used the HLVa questionnaire to assess vaccine literacy functional and interactive-critical skills and its associated demographic factors in these patients. It is important to underline that previous studies conducted in patients with autoimmune diseases have used tools to assess general health literacy but not exactly to evaluate vaccine literacy (Katz et al., 2021; Maheswaranathan et al., 2020; Wu et al., 2020). Future researchers should consider using Online Photovoice (OPV) to further research this topic. OPV is one of the most recent and effective innovative qualitative research methods that gives opportunities to the participants to express their own experience with as little manipulation as possible if at all, compared to traditional quantitative methods (Tanhan & Strack, 2020).

The findings of this study highlight the need to design and implement educational programs to improve vaccine literacy among autoimmune disease patients. Such programs, that can be implemented through cooperation between health-care providers and medical staff in different locations including hospitals, health centers and educational or work environment, might allow patients to identify reliable and timely information from credible sources. This may mitigate the negative consequences of misinformation on COVID-19 vaccines. In addition, our findings, which may serve as preliminary considerations, support the need for consideration of certain sociodemographic factors underlying the level of vaccine literacy, focusing on the role of area of residence, civil status, socioeconomic status and educational attainment. Vaccine literacy programs must focus on patients living in rural areas, single or living-in unit, with middle/low socioeconomic status and with lower education in order to reduce existing COVID-19 vaccine literacy disparities. Health care professionals should incorporate initiatives to increase vaccine literacy among autoimmune disease patients into the daily health care services they provide (Voigt-Barbarowicz & Brütt, 2020).

In conclusion, this study revealed that the level of vaccine literacy for functional and interactive-critical scales were medium in patients with autoimmune diseases. Area of residence, civil status and socioeconomic status represented determinants of vaccine literacy interactive-critical scale, and educational attainment also contribute to vaccine literacy functional scale. Insight into these factors is required to ensure an optimal vaccine literacy level in patients with autoimmune diseases.

### Implications

Public health officials should consider area of residence, civil status, socioeconomic status and educational attainment as determinants of vaccine literacy in patients with autoimmune diseases. These factors might be considered in any plans and interventions to improve vaccine literacy and therefore, to increase vaccine trust and acceptability in patients with systemic autoimmune diseases.

## Supplementary Information

Below is the link to the electronic supplementary material.

Supplementary file1 (DOCX 25 kb)

## Data Availability

The datasets generated during and/or analysed during the current study are available from the corresponding author on reasonable request.
