# Supplementary material for: COVID-19 vaccine literacy in patients with systemic autoimmune diseases
Source: Curr Psychol. 2022 Jan 18:1–16. Online ahead of print. doi: 10.1007/s12144-022-02713-y (PMC8764502; doi:10.1007/s12144-022-02713-y)
Supplement: Supplementary file 1 — Supplementary file1 (DOCX 25 kb) [file 12144_2022_2713_MOESM1_ESM.docx]

**Supplementary Table 1.** Kolmogorov–Smirnov test for the variables included in this study.

|  | Kolmogorov–Smirnov test |
| --- | --- |
| **Sociodemographic characteristics** | **<**0.001 |
| Gender | **<**0.001 |
| Age groups | **<**0.001 |
| Country of residence | **<**0.001 |
| Area of residence | **<**0.001 |
| Civil Status | **<**0.001 |
| Socioeconomic status | **<**0.001 |
| Educational attainment | **<**0.001 |
| Occupational status | **<**0.001 |
| **VL functional skills** | **<**0.001 |
| 1. Did you find that the material as a whole (texts and/or images) was difficult to read? | **<**0.001 |
| 2.Did you find words you didn’t know? | **<**0.001 |
| 3. Did you find that the texts were difficult to understand? | **<**0.001 |
| 4. Did you need much time to understand them? | **<**0.001 |
| 5. Did you or would you need someone to help you understand them? | **<**0.001 |
| **VL interactive/critical skills** | **<**0.001 |
| 6. Have you consulted more than one source of information? | **<**0.001 |
| 7. Did you find the information you were looking for? | **<**0.001 |
| 8. Did you understand the information found? | **<**0.001 |
| 9. Have you had the opportunity to use the information? | **<**0.001 |
| 10. Did you discuss what you understood about vaccinations with your doctor or other people? | **<**0.001 |
| 11. Did you consider whether the information collected was about your condition? | **<**0.001 |
| 12. Have you considered the credibility of the sources? | **<**0.001 |
| 13. Did you check whether the information was correct? | **<**0.001 |
| 14. Did you find any useful information to make a decision on whether or not to get vaccinated? | **<**0.001 |
| **Attitudes and perceptions** | **<**0.001 |
| 1. Do you think the vaccines developed so far are safe? | **<**0.001 |
| 2. Do you think they are efficacious? | **<**0.001 |
| 3. Do you think they overlap, regardless of the production technique used? | **<**0.001 |
| 4.Do you intend to get vaccinated against COVID-19? | **<**0.001 |
| 5. If you could, would you choose which vaccine to take? | **<**0.001 |
| 6. Will the Government be able to offer the vaccine against COVID-19 for everyone for free? | **<**0.001 |
| 7. Would you pay a fee to be vaccinated? | **<**0.001 |
| 8. Should vaccination against COVID-19 be made mandatory for everyone? | **<**0.001 |
| 9. Should vaccination against COVID-19 be made compulsory for the most | **<**0.001 |
| at-risk groups? | **<**0.001 |
| 10. Do you think children should be vaccinated too? | **<**0.001 |
| 11.Have you been vaccinated against flu last season? | **<**0.001 |
| 12. Did you want to be vaccinated against the flu, but you couldn’t? | **<**0.001 |
| 13. Have you been recently vaccinated and/or do you intend to be vaccinated soon against other infectious diseases, in addition to seasonal influenza and COVID-19? | **<**0.001 |
| 11.Have you been vaccinated against flu last season? | **<**0.001 |
| 12. Did you want to be vaccinated against the flu, but you couldn’t? | **<**0.001 |
| 13. Have you been recently vaccinated and/or do you intend to be vaccinated soon against other infectious diseases, in addition to seasonal influenza and COVID-19? | **<**0.001 |
| **Beliefs** | **<**0.001 |
| 1. I am not favorable to vaccines because they are unsafe | **<**0.001 |
| 2. There is no need to vaccinate because natural immunity exists | **<**0.001 |
| 3. Vaccines are effective at preventing diseases | **<**0.001 |
| 4. I generally do what my health care professional recommends | **<**0.001 |
| 5. Getting myself vaccinated for COVID-19 would be a good way to protect myself against infection | **<**0.001 |
| 6. My family and friends would probably think that getting a COVID-19 vaccine is a good idea | **<**0.001 |
| 7. To protect public health, we should follow government guidelines about vaccines | **<**0.001 |
| 8. Patients with risk factors should be the first ones to get the COVID-19 vaccine when available | **<**0.001 |

VL: vaccine literacy
